# Global Trends of Usage of Chlorophyll Fluorescence and Projections for the Next Decade

**DOI:** 10.34133/2020/6293145

**Published:** 2020-12-08

**Authors:** Alonso Zavafer, Leen Labeeuw, Cristian Mancilla

**Affiliations:** ^1^Research School of Biology, The Australian National University, Canberra, ACT, Australia; ^2^University of Technology Sydney, Climate Change Cluster, Faculty of Science, Sydney, NSW 2007, Australia; ^3^Independent Researcher, La Florida, Chile

## Abstract

Chlorophyll fluorescence is the most widely used set of techniques to probe photosynthesis and plant stress. Its great versatility has given rise to different routine methods to study plants and algae. The three main technical platforms are pulse amplitude modulation (PAM), fast rise of chlorophyll fluorescence, and fast repetition rate. Solar-induced fluorescence (SIF) has also gained interest in the last few years. Works have compared their advantages and their underlying theory, with many arguments advanced as to which method is the most accurate and useful. To date, no data has assessed the exact magnitude of popularity and influence for each methodology. In this work, we have taken the bibliometrics of the past decade for each of the four platforms, have evaluated the public scientific opinion toward each method, and possibly identified a geographical bias. We used various metrics to assess influence and popularity for the four routine platforms compared in this study and found that, overall, PAM currently has the highest values, although the more recent SIF has increased in popularity rapidly during the last decade. This indicates that PAM is currently one of the fundamental tools in chlorophyll fluorescence.

## 1. Introduction

Chlorophyll fluorometry is one of the most popular sets of techniques to probe photosynthesis worldwide [1] and has become one of the fundamental tools in phenotype-driven research in plant breeding (plant phenomics) [2]. This type of fluorometry comprises a set of techniques that focus on the light emitted by the fluorophore photosynthetic pigment chlorophyll *a* [1, 3]. The theoretical basis for the use of chlorophyll fluorescence as a phenotyping technique depends on quenching analysis that relates to the state of the photosynthetic electron transport reactions [4, 5]. When the pool of acceptors is saturated, an increase in the fluorescence intensity is observed, and the opposite scenario occurs when the fluorescence intensity decreases [4, 5]. Chlorophyll fluorometry originated in the late 1930s with Kautsky—after whom the effect of the rise of the fluorescence was named [6]. From the 1960s to the 1980s, efforts done by biophysicists worldwide deconvoluted several properties of the signal [6, 7], which became the basis for all routine methods used nowadays.

Chlorophyll fluorescence is an intense signal (with a yield of around 2% of the absorbed light) and requires little sample preparation [8], making it one of the most attractive techniques in plant phenomics. Several methods explore different properties of photosynthetic material via fluorescence, such as structure (confocal and epifluorescence [9]), energy transfer (time correlated to single-photon counting, fluorescence lifetime microscopy, or just steady-state fluorescence [10, 11]), and charge recombination reactions (delayed fluorescence [12, 13]). There are, however, approaches that could be considered routine methods and are key tools in contemporary phenomic research. According to Kalaji et al. [1], three major classes of methods could be recognized regarding the capacity to probe photosynthetic reactions: single turnover flashes (STF), the fast induction of chlorophyll fluorescence (prompt fluorescence), and photosynthetic steady-state quenching analysis (henceforth quenching analysis) [1]. STF uses strong flashes of light that reduce the acceptor side of photosystem II (PSII), providing information primarily on the electron transport inside PSII. Prompt fluorescence offers information on the redox state of the photosynthetic electron transport [1]. Quenching analysis measures, overall, several aspects of photosynthetic reactions, such as electron transport rates, photoprotection, and the Calvin-Benson cycle [1]. These three routine approaches can be implemented in three main general types of instruments depending on the way fluorescence is induced: short flash excitation, strong continuous excitation, and emission during stable photosynthetic activity [1]. While these approaches could be combined, all these types of methods and instruments are commonly implemented in three popular technical platforms: pulse amplitude modulation (PAM [14]), the fast polyphasic rise of the induction by continuous excitation (addressed here as OJIP [13, 15, 16]), and the fast repetition rate fluorometry (FRR [17, 18]).

PAM is a platform that uses modulated light allowing the exclusion of other environmental lights [14]. The modulated light has low intensity and has a negligible effect of photosynthetic reactions [14]. In this way, PAM can be implemented for quenching analysis in steady-state conditions by the superimposition of other nonmodulated light to control photosynthesis without affecting the emitted fluorescence [14]. It is common, then, to refer the term PAM to quenching analysis and the emission of nonmodulated light during stable photosynthetic activity methods. In the case of OJIP, the measurement is done by using direct continuous excitation and the OJIP acronym refers to the inflection points of the chlorophyll fluorescence induction curve (at the following time points: 10 *μ*s, 2 ms, 30 ms and peak value), which is the most common measured feature. It is widely accepted that these inflection points relate to the redox state of the electron transport chain and occur during the first microseconds to seconds of continuous illumination [15]. As most authors and instruments that measure the OJIP curve use strong continuous excitation, OJIP in this work will refer only to such instruments and methods. It is important to point out that the polyphasic curve could be measured using PAM as well, but authors refer to the inflection points differently (O, I1, I2, and P) and in these works will not be considered as part of the OJIP technical platform [19, 20]. FRR uses several consecutive fast flashlets as probing light that saturate in discrete quantities the photosynthetic electron acceptors [17]. FRR could be considered a hybrid platform since it allows single and multiple turnover excitation and studies stable photosynthetic activity. These three technical platforms have become quite popular in several fields of plant and algal research due to their ease in measuring [1, 3], the availability of commercial instruments [2], and the portability of these devices [21–23]. In addition to these three prevalent platforms, solar- (sun-) induced fluorometry (SIF) has gained popularity over the last decade as a platform to analyse responses under “real-world conditions” in a remote fashion, such as large areas of crops and forests [24, 25]. The principle of SIF involves detecting sunlight-induced fluorescence by estimating the difference of intensity between two wavelengths within the near infrared and correcting it against the sunlight spectrum [25]. While methodological aspects are well established for SIF [26], the *why and how* of this signal correlation to photophysiological processes remains under scrutiny [27].

While these platforms are generally acknowledged as complementary to each other, heated debates on the interpretation of chlorophyll fluorescence signal are common in the field of chlorophyll fluorescence [5, 6]. Which of these four platforms is the most popular and influential worldwide? Is there a particular platform of choice for each field of research? Does the general plant scientific community trust one platform over the rest? All these questions could be studied by bibliographic metrics to assess popularity (here understood as a general acceptance) and influence (citation metrics) of the four given platforms. While answers to these questions do not necessarily reveal which platform is objectively best, they can describe the *status quo* in the field of photosynthesis research during this decade. In order to study this, we use metrics such as the *h*-index for each platform, the number of published articles per platform, and the number of citations per platform. In addition, we explore whether there is a bias in country usage of each platform, type of journal, and impact (via CiteScore). By integrating all this information, we will discuss the popularity of each platform.

## 2. Materials and Methods

### 2.1. Data Gathering

Publications per year, number of citations per year and per article, and name of the journal per fluorometry platform were obtained for the decade 2009-2019 via the citation tracker and database Scopus (Elsevier, Amsterdam, NL). To find the highest possible number of publications per platform during the last ten years, several combinations of keywords were added until the number of articles did not increase further.  Table 1 indicates the combination of keywords used in all available fields of the Scopus browser (such as article title and abstract). The search was done from October 6th to 9th, 2019. The search was filtered so that only research articles in peer-reviewed journals were included. Articles published in languages other than English were eliminated through a series of iterations. In this sampling, we have not eliminated self-citations. No distinction was made between research articles, opinions, reviews, etc. The impact of the journal was obtained from CiteScore. To obtain the number of articles per journal, the “CTOExport” file obtained in Scopus was filtered and only the column of “Journal name” was considered.

### 2.2. Filtration of Data

We compared the list of articles between the four platforms and estimated the number of articles that were repeated in more than one list. The number of articles published per journal was estimated by frequency counts.

### 2.3. Parameters Used in This Work

We have defined the *popularity of a platform* as the general acceptance or approval of a given platform. To assess this, we have used the following criteria: first, the total number of published articles in a decade that did not use more than one platform (*A*_Tot_) was assessed to estimate the popularity of the platform; second, the publication rate in a decade (*k*_Pub_), which was estimated by linear regression or exponential fit obtained from the time course of the number of published articles per year—for the equations used, please refer to the legend of each figure. We estimated the relative popularity per country using the sum of the number of articles published in a country (determined by the location of the institution an author is ascribed to, *P*_*C*_). To establish the economic context of each country, we considered two types of countries: developed or developing, based on the “World Economic Situation and Prospects (WESP)” published by the United Nations [28]. Also, to establish if there is a correlation between the popularity of the platform in a country and scientific domain of the country, we referred to the database published by SCImago Journal & Country Rank of the Spanish National Research Council. This information was checked on October 10th, 2019, and focuses on biology and plant sciences.

We have defined the *influence of a platform* as the capacity of a platform to make an effect on published works by means of total citations and acceptance of its articles in high CiteScore journals (CiteScore bias). To assess citations, two metrics were used. The first is *h*-index (*h*), which is calculated by counting the number of publications in which a platform has been cited by other works at least the same number of times. We also use a parameter that we name “*Σ*_*c*_*t*__”, which stands for the total number of citations of an article weighed by its age (*c*_*t*_) and divided by the number of articles to correct against the corpus size or “*v*” (see Equation (1)). For example, the *c*_*t*_ of an article with 950 citations published in 2015 would be calculated as 950 citations per 4 years = 237.5 citations/year. If a platform has a sum of *c*_*t*_ equal to 230 citations per year but the corpus size is 30 articles, then *Σ*_*c*_*t*__ equals 7.6 citations per year per article. To estimate the impact score (CiteScore or *C*), we calculated the weighted average impact score of the journals in which at least ten articles have been published during the last decade—we will term this parameter *J*_10_. The weight is done by:

(1) $$\begin{array}{c} J_{10}=\overset{n}{\underset{y=1}{\sum }}C_{n}*\frac{a_{n}}{a_{10}}, \end{array}$$

where *a*_*n*_ is the number of articles in a journal and *a*_10_ is the total number of articles in journals with at least ten articles of a given platform. The benchmark of at least ten articles is based on Callaway [29], who showed that the articles with at least ten citations are the ones that contribute to the citations of a journal.

To condense all measurements, we proposed an “Influence Index per Article” calculated as follows:

(2) $$\begin{array}{c} i=\left(\left(\Sigma_{c_{t}}\right)*h\right)*{A_{\text{Tot}}}^{-1}. \end{array}$$

The reason behind using two citation-based metrics, such as *Σ*_*c*_*t*__ and the *h*-index, is that both platforms explain different aspects of the citations dynamics. The first component of the equation allows us to normalize citations to correct citation bias and *h*-index. However, *h*-index is highly dependent on the corpus size, and comparing two platforms with different corpora would induce bias. Therefore, the product of these two citation parameters is normalized to the weighted CiteScore bias. This would correct the effect that authors may prefer to cite works accepted in higher CiteScore journals than those with lower impacts.

### 2.4. Processing of the Data

The data were processed statistically and graphically with the following software and packages: Microsoft Excel (Microsoft Excel (Internet) 2018, Microsoft Corporation; available from https://office.microsoft.com/excel), Origin(Pro) (version 2018b; OriginLab Corporation, Northampton, MA, USA), SPSS 25 (IBM SPSS Statistics for Windows, version 25.0, released in 2017; IBM Corp., Armonk, NY), and Python 2.7 and R libraries such as NumPy and Matplotlib.

## 3. Results

### 3.1. The Most Popular Platform for Chlorophyll Fluorescence of the Last Decade Is PAM

**Figure 1:**
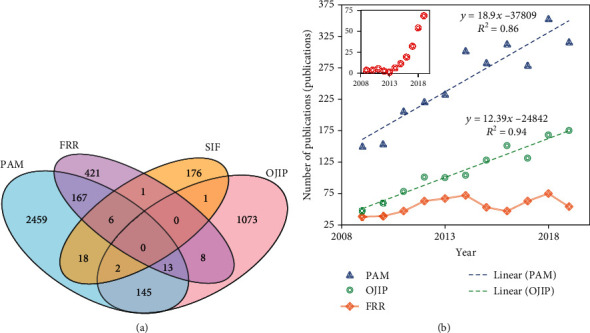
Popularity of different fluorometric methods based on publication count of the last ten years. (a) Venn diagram of the total number of exclusive publications (*A*_Tot_). (b) Trends of publications per year for each method. Linear equations were used for PAM and OJIP (*y* = *kx* + *b*, where *y* is the number of publications per year, *x* is the year, *b* is the offset based on the previous decade, and *k* is the rate constant) while exponential fitting was used for SIF (*y* = *ekx*, where *y* is the number of publications per year, *x* is the year, and *k* is the rate constant).

The publication count for PAM, OJIP, FRR, and SIF shows that, during the last ten years (2009-2019), there have been a total of 4,490 articles for our sample for all the four platforms. In Figure 1(a), a Venn diagram displays the number of articles per platform. PAM counts 2,459 exclusive publications (55%), OJIP counts 1,073 exclusive publications (24% of the total publications), FRR counts 421 exclusive publications (9%), and SIF counts 176 exclusive publications (4% of the total publications). Publications that mention more than one platform account for nearly 8%. Based on this metric, PAM would be the most popular, doubling OJIP in popularity. Based on publications per year (Figure 1(b)), PAM publications have increased linearly over a decade at a rate of 18.9 ± 2.5 (*R*^2^ = 0.84) publications per year. OJIP increased linearly at a rate of 12.1 ± 1 publications per year (*R*^2^ = 0.94). Although FRR increased its popularity between 2009 and 2013, the platform has remained on a steady publication number around 80 publications per year with no significant increase since then—we could not find a model that fits adequately. It was surprising to realise that SIF has increased its popularity exponentially with a doubling time of 3.1 ± 0.001 per year (*R*^2^ = 0.99) since 2013. If the trend were to continue in this manner, SIF would be the most popular platform of chlorophyll fluorescence by 2025 (projected data in Figure S1).

### 3.2. PAM, FRR, and SIF Are More Popular in Developed Countries, While OJIP Is Popular in Developing Countries

**Figure 2:**
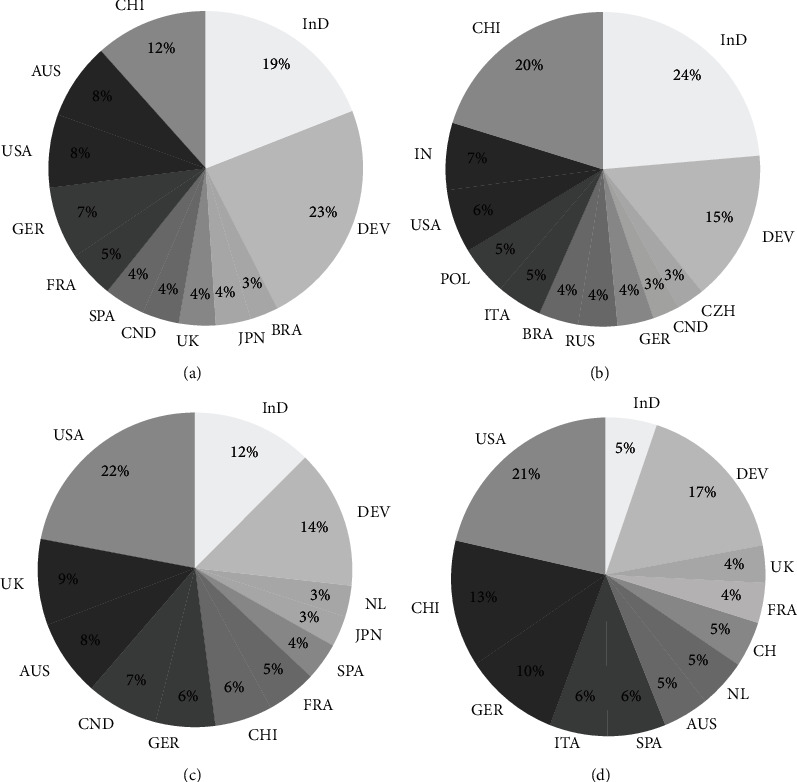
Relative popularity of different fluorometric methods based on publication count during the last ten years by country. (a) PAM, (b) OJIP, (c) FRR, and (d) SIF. Total ascribed institutions per country were normalized to the total institutions worldwide for each method. Developed countries with less than 3% of publications were summed together and are listed as “Dev”. Developing countries with less than 3% of publications were summed together and are listed as “InD”.

To verify whether there was a trend related to geographical regions, we use the *P*_*C*_ parameter. For PAM entries, we have identified 4,266 entries from 72 countries. Half of them come from eight countries: China—495, Australia—334, United States—332, Germany—308, France—208, Spain—187, Canada—157, United Kingdom—155, Japan—151, and Brazil—128. In total, for PAM users, 66% come from developed countries, which shows that PAM fluorometry is much more popular in developed countries (Figure 2(a)).

Amongst the OJIP entries, we have identified authors from 77 countries. Half of them come from seven countries: China—364, India—128, United States—116, Poland—90, Italy—85, Brazil—74, and Russia—72 (Figure 2(b)). OJIP is more popular in developing countries as 60% of the authors' institutions are localized in these countries. In fact, the top three most cited articles dealing with OJIP during the last ten years come from authors based in developing countries.

In the case of FRR, the 701 entries were published by 1,232 authors from 59 countries. Half of them come from five countries: the US—269, UK—109, Australia—97, Canada—90, and Germany—75. For FRR, 82% of the entries of ascribed institutions come from developed countries, which means that FRR is popular almost exclusively in developed countries.

Finally, 611 entries were found for SIF works. SIF is less spread worldwide, with only 43 countries represented, and half of them come from four countries: the US—131, China—81, Germany—59, and Italy—36. For SIF, 77% of publications come from developed countries.

**Figure 3:**
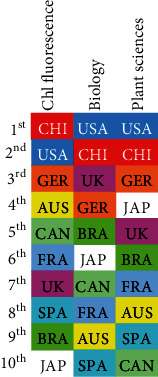
Comparison of the top-ten ranking of chlorophyll fluorescence by country versus SCImago rankings for biology and plant sciences. The heading of every column shows the ranking type: “Chl fluorescence” is estimated from data in Figure 2 and corresponds to chlorophyll fluorescence overall; “Biology” and “Plant sciences” refer to the SCImago ranking calculated on absolute publication count. The colour code is indicative of the country, and the country abbreviations used are as follows: Australia, AUS (yellow); Brazil, BRA (green); Canada, CAN (light green); China, CHI (red); France, FRA (light blue); Germany, GER (orange); Japan, JAP (grey); Spain, SPA (cyan); United Kingdom, UK (pink); and the United States of America, USA (blue).

We then summed the total number of entries for each platform together to estimate the scientific output using chlorophyll fluorescence per country. This output was ranked in a top-ten list and compared to the SCImago ranking in the field of biology and plant sciences (Figure 3). We found that the country with most authors using chlorophyll fluorescence is China, which ranks second in scientific domain per publications on both biology and plant sciences. The USA was the second user of chlorophyll fluorescence and first in the SCImago ranking of both fields. Germany ranked third in chlorophyll fluorescence, which correlates to being the third producer in plant science publications. Australia was, remarkably, the fourth main user, while it ranks 9th in biology and 8th in plant science publications. Similarly, Canada was the 5th main user of fluorometry but the 7th and 10th producer of biological and plant science papers. The rest of the countries did not correlate well with the SCImago ranking. Finally, it seems remarkable that Japan, which is the fourth producer of plant science papers, ranked last in the top-ten users of chlorophyll fluorescence.

### 3.3. Influence Based on Weighted Citations Shows That PAM Is the Most Influential, but SIF Articles Are the Most Cited per Article

**Figure 4:**
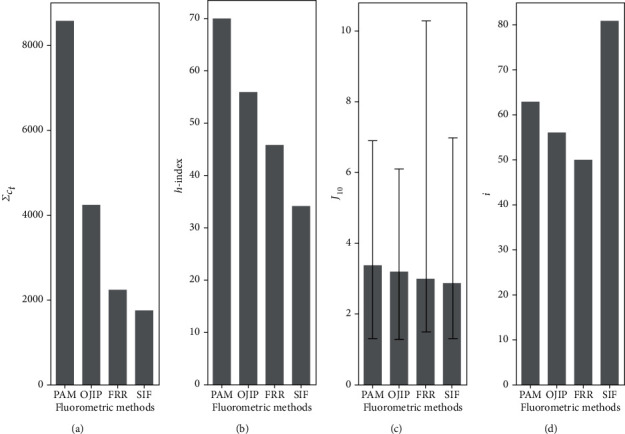
Relative influence of different fluorometric methods based on citation metrics during the last ten years and on CiteScore. (a) Adjusted citations per article per year (*Σ*_*c*_*t*__), (b) *h*-index, (c) average weighted CiteScore (*J*_10_) with error bar denoting absolute maximum and minimum impact factor, and (d) influence index (*i*).

Besides popularity, the influence of each fluorometric platform can be assessed via citation scores. To do so, we compared the weighted total citations by time, *Σ*_*c*_*t*__ (Figure 4(a)). Coinciding with the popularity assessment, *Σ*_*c*_*t*__ was the highest for PAM with 8,595 citations, followed by 4,246 citations for OJIP, 2,242 citations for FRR, and 1,764 citations for SIF. The observed *Σ*_*c*_*t*__ pattern also coincides with the absolute citations per platform as shown in Figure S2. Interestingly, if adjusted to the corpus size (*v*), SIF has the highest score of 8.1 citations per article, followed by FRR with 3.7 citations per article, OJIP with 3.4 citations per article, and PAM with 3.1 citations per article. This calculation shows that SIF has gained relevance both about popularity and citations per published article. Please note that here we have used the absolute corpus size and not just the exclusive articles as done in Figure 1(a).

### 3.4. The *h*-Index Shows That PAM Is the Most Influential and OJIP the Second Most Influential

When the *h*-index is used instead to assess influence (Figure 4(b)), we observe that PAM is the highest with a value of 70, followed by OJIP with a value of 56, then a value of 46 for FRR, and 34 for SIF. This pattern coincides with the one observed for *Σ*_*c*_*t*__ (Figure 4(a)).

### 3.5. The CiteScore Factor Bias per Platform Is Nearly the Same for Each of the Four Platforms

We used the *J*_10_ —which reflects the weighted average CiteScore for each platform. Note that we considered journals that have published at least ten articles on a single platform during the last decade. We observed that the *J*_10_ for PAM was 3.4 (absolute min 2.1 and max 3.5 CiteScore factor), OJIP 3.2 (absolute min 1.9 and max 2.9 CiteScore factor), FRR 3 (absolute min 1.5 and max 7.3 CiteScore factor), and SIF 2.9 (absolute min 1.6 and max 4.1 CiteScore factor).

For PAM, we found 63 journals that published ten or more articles, which equals 1,655 (59%) of the PAM publications during the last decade. The two specialized journals in photosynthesis (*Photosynthesis Research* and *Photosynthetica*) have the highest publications related to PAM with a total of 174 between the two (see Figure S3). The *Journal of Applied Phycology* also reached a close number of publications with 71 publications. PAM fluorometry seemed popular in a broad range of topics, such as general plant biology, algae research, and marine biology (Figure S3).

For OJIP fluorometry, 22 journals have published ten or more OJIP-related articles. This comprises 639 publications—51% of the total. In the same fashion as PAM, two of the most popular journals for OJIP fluorometry, *Photosynthesis Research* and *Photosynthetica*, have published 158 between the two. The rest of the publications have mostly appeared on general plant biology journals (Figure S4).

For FFR fluorometry, we found that 16 journals have published ten or more articles. This comprises 279 publications—40% of the total. *PLoS One*, a nonspecialized megajournal, has the highest number of articles with 36 and *Photosynthesis Research* was second with 31. In the case of FRR, there is a tendency to publish in marine biology journals—13 out of the 16 journals fall within this category (Figure S5).

In the case of SIF fluorometry, we found that only three journals have published ten or more articles. This comprises 91 publications—44% of the total in this decade. It is clear here that the tendency of SIF is to be used in journals focused on remote sensing—the top two journals were *Remote Sensing of Environment* and *Remote Sensing*.

When all the metrics were condensed to calculate the parameter *i* (see equation (2)), we observed that SIF had the higher value with 81, followed by PAM with 63, OJIP with 56, and FRR with 50.

## 4. Discussion

In this work, PAM, OJIP, FRR, and SIF were analysed based on their popularity (number of publications, rates of publication, and articles per country) and influence (citation numbers, *h*-factor, and CiteScore factor) within the last decade (2009 to 2019). We selected this period as it represents the current state of the field about chlorophyll fluorescence.

Popularity based on the number of published articles indicates that PAM is the most popular platform, followed by OJIP, FRR, and then SIF. This could be explained by the notion that PAM signals are simpler to interpret because this platform employs modulated light to detect the photosynthetic signals [1, 3, 14]. As PAM instruments only detect modulated light, all other lights, such as ambient light, do not interfere with the detection of the platform. On the other hand, OJIP and FRR are platforms that remain controversial [1, 3, 14, 30], as several theoretical frameworks have been postulated to explain the measured signals. For example, OJIP has four main theoretical frameworks to analyse the OJIP curve [5, 6, 15, 31–34] and the use of differing mathematical models may have an impact on the number of users of this platform [6, 32, 33]. Discrepancies in the photosynthetic quantum yield obtained through FRR and PAM (the most popular platform) could cause an impact on the impression that users may have about the platform [17, 18, 30]. FRR has been used primarily in oceanographic works [35], as we have observed in this work, so it is somewhat of a niche platform. Finally, there is no surprise that SIF is the last in popularity, as this is the most recently developed of the four technical platforms [24].

Nevertheless, popularity based solely on publication counts may not represent the actual popularity of each platform. The publication rate per year shows that, while SIF is the newest development, interest on it is growing quite fast and it is likely that, if the trend continues, by 2025 it will become the most popular platform of fluorometry. One should bear in mind that this trend may be an effect of the initial interest in the field, so it may change in the future. However, the elevated necessity of studying crops and forests remotely [26], in part due to the impact of climate change [36], make us hypothesize that the trend will continue. On the other hand, the trends also show that the corpus of PAM is likely to keep growing at a steady rate during the next decade. Neither OJIP nor FRR are likely to become the most popular platform unless breakthroughs on these technologies appear in the next decade.

A regional analysis of the popularity of fluorometry also showed interesting data on the use of each platform by country. OJIP fluorometry, for example, is mostly popular in developing countries. There could be two possible explanations for this phenomenon. First, due to the simplicity of OJIP fluorometers (which only consist of a detector, LED, and data acquisition devices) [37], their cost has been historically lower than PAM fluorometers. PAM fluorometers originally required lock-in amplifiers (used to filter the modulated fluorescence) [38, 39], the cost of which ranges from hundreds to thousands of USD [40], what would increase their retail price, while OJIP fluorometers are assembled for as low as 100 USD [37]. A second explanation for the popularity of OJIP fluorometers in developing countries is the well-known educational campaigns of Prof. Strasser [41, 42] and Prof. Govindjee [43–45] (main developers of the popular JIP test) in developing countries (particularly China and India) during the last 30 years. However, this tendency may change due to low-cost commercial PAM instruments (such as Photon Systems Instruments' FluorPen [46] or the multisensor device PhotosynQ [47]). Another interesting observation about regional popularity is that FRR is primarily used in developed countries. This may also be explained due to instrument costs, as with PAM, but this situation could change also thanks to the development of open-source FRR instrumentation [48], which should make this technology more accessible in developing countries. Furthermore, China is likely the main user of most fluorometric platforms, except for FRR. In the case of FRR, the USA [18], UK [49], and Canada [50, 51] sum up nearly 40% of the total users worldwide, which correlates to the fact that most of the instrumentation is manufactured in these countries.

PAM seems to be the most influential platform when the total number of citations, citations weighted by the age of its articles, and *h*-index are considered. All this makes us hypothesize that the global scientific community is more inclined toward PAM. This platform is followed by OJIP and FRR in influence according to the aforementioned metrics. However, when this data is corrected against the corpus size, we can see that SIF is used in articles that hold a higher influence overall, followed by PAM, OJIP, and FRR. SIF has been heavily promoted for use in agriculture, which may account for its high influence [52]. The analysis based on the CiteScore indicated that the four platforms are rather similar.

This study is intended to help newcomers in the field understand the relative popularity and influence of each of these platforms, which may guide them in their decision-making. This report can help instrument manufacturers to decide on which markets to focus, to cover unattended geographical areas, and to take advantage of the current trends in the use of chlorophyll fluorescence. We acknowledge that some limitations may exist in our study: while we investigated a comprehensive sample of articles for each of the platforms, the search algorithms used in searching the Scopus database do not differentiate to which degree a platform was used within each article and it would be impossible to manually assess the more than 4,900 papers that comprise our sample.

## 5. Conclusions

In this work, we have examined the popularity and influence of four of the most common platforms of chlorophyll *a* fluorometry. Our analysis showed that while PAM is the most popular and influential platform, SIF is gaining relevance and it is likely to surpass PAM fluorometry regarding both popularity and influence within the next decade. OJIP is currently the second most popular platform, but it is primarily used in developing countries. Finally, the observed trends for FRR indicate that this platform may remain a niche research platform.

## Figures and Tables

**Table 1:** Keywords used in Scopus to find relevant articles related to a fluorometric method. Exclusion of languages other than English had to be done in addition to the instruction LIMIT-TO English in some cases, as the search yielded false-positive results in other languages.

Method	Keyword instructions
PAM	((ALL(“Pulse Amplitude Modulation” OR “PAM”) AND ALL(chlorophyll)) AND PUBYEAR >2008 AND (EXCLUDE (PUBYEAR,2020)) AND (LIMIT-TO (LANGUAGE,“English”)) AND (LIMIT-TO (SRCTYPE,“j”)) AND (EXCLUDE (LANGUAGE,“Spanish”) OR EXCLUDE (LANGUAGE,“Chinese”) OR EXCLUDE (LANGUAGE,“Hungarian”)))

OJIP	(ALL(“OJIP” and “fluorescence”) AND PUBYEAR >2008 AND (LIMIT-TO (LANGUAGE,“English”)) AND (LIMIT-TO (SRCTYPE,“j”)) AND (EXCLUDE (LANGUAGE,“Spanish”) OR EXCLUDE (LANGUAGE,“Chinese”) OR EXCLUDE (LANGUAGE,“Hungarian”)))

FRR	(ALL(“fast repetition rate” and “fluorescence”) AND PUBYEAR >2008 AND (LIMIT-TO (LANGUAGE,“English”)) AND (LIMIT-TO (SRCTYPE,“j”)) AND (EXCLUDE (LANGUAGE,“Spanish”)))

SIF	(ALL(“Solar-induced fluorescence” and “chlorophyll”) AND (LIMIT-TO (LANGUAGE,“English”)) AND (LIMIT-TO (SRCTYPE,“j”)) AND (LIMIT-TO (PUBYEAR,2019) OR LIMIT-TO (PUBYEAR,2018) OR LIMIT-TO (PUBYEAR,2017) OR LIMIT-TO (PUBYEAR,2016) OR LIMIT-TO (PUBYEAR,2015) OR LIMIT-TO (PUBYEAR,2014) OR LIMIT-TO (PUBYEAR,2013) OR LIMIT-TO (PUBYEAR,2012) OR LIMIT-TO (PUBYEAR,2011) OR LIMIT-TO (PUBYEAR,2010) OR LIMIT-TO (PUBYEAR,2009)))

## Abbreviations

PAM: Pulse amplitude modulation
FRR: Fast repetition rate
SIF: Solar-induced fluorescence.

## Supplementary Materials

Supplementary Materials Figure S1: projections of the increase of three fluorometric methods based on the trends observed in the decade 2009-2019. Figure S2: comparison of the absolute citations per method. Figure S3: scientific journals with at least 10 published PAM articles. Figure S4: scientific journals with at least 10 published OJIP articles. Figure S5: scientific journals with at least 10 published FRR articles.
