# Supplementary material for: Global Trends of Usage of Chlorophyll Fluorescence and Projections for the Next Decade
Source: Plant Phenomics. 2020 Dec 8;2020:6293145. doi: 10.34133/2020/6293145 (PMC7869938; doi:10.34133/2020/6293145)
Supplement: Supplementary Materials — Figure S1: projections of the increase of three fluorometric methods based on the trends observed in the decade 2009-2019. Figure S2: comparison of the absolute citations per method. Figure S3: scientific journals with at least 10 published PAM articles. Figure S4: scientific journals with at least 10 published OJIP articles. Figure S5: scientific journals with at least 10 published FRR articles. [file 6293145.f1.pdf]

## Supplementary material

# Global trends of usage of chlorophyll fluorescence and projections for the next decade

Alonso Zavafer[a], Leen Labeeuw[b], Cristian Mancilla[c]

[a] Research School of Biology, the Australian National University, Canberra, ACT, Australia

[b] University of Technology Sydney, Climate Change Cluster, Faculty of Science, Sydney, NSW 2007, Australia

[c] Los Lagos 9855, La Florida 8300500, Chile

Email – Alonso Zavafer: alonso.zavaleta@anu.edu.au

Email – Leen Labeeuw: leen.labeeuw@uts.edu.au

Email – Cristian Mancilla: cristian.mancilla@umce.cl

Author for correspondence: Alonso Zavafer

Telephone: +61 2 6172 1450

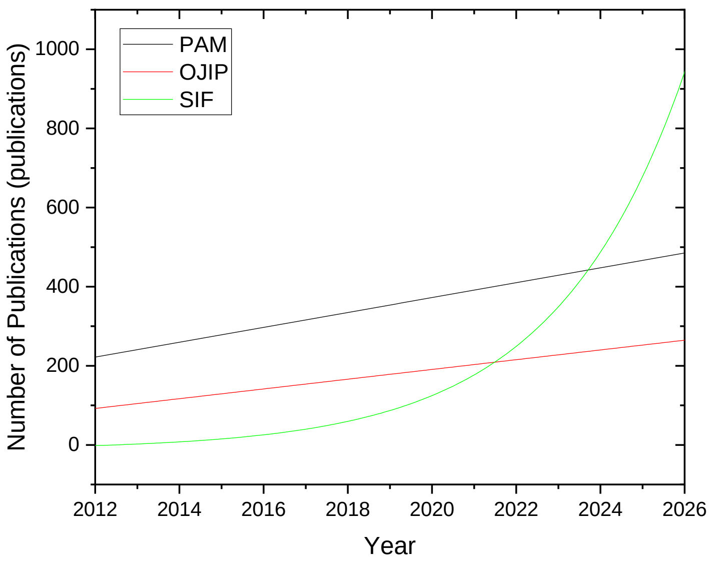


Figure S1: Projections of the increase of three fluorometric methods based on the trends observed in the decade 2009-2019.

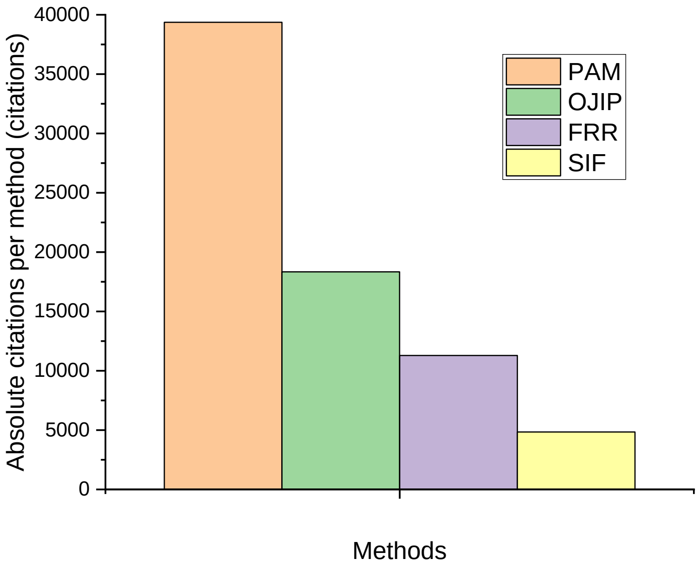


Figure S2: Comparison of the absolute citations per method.

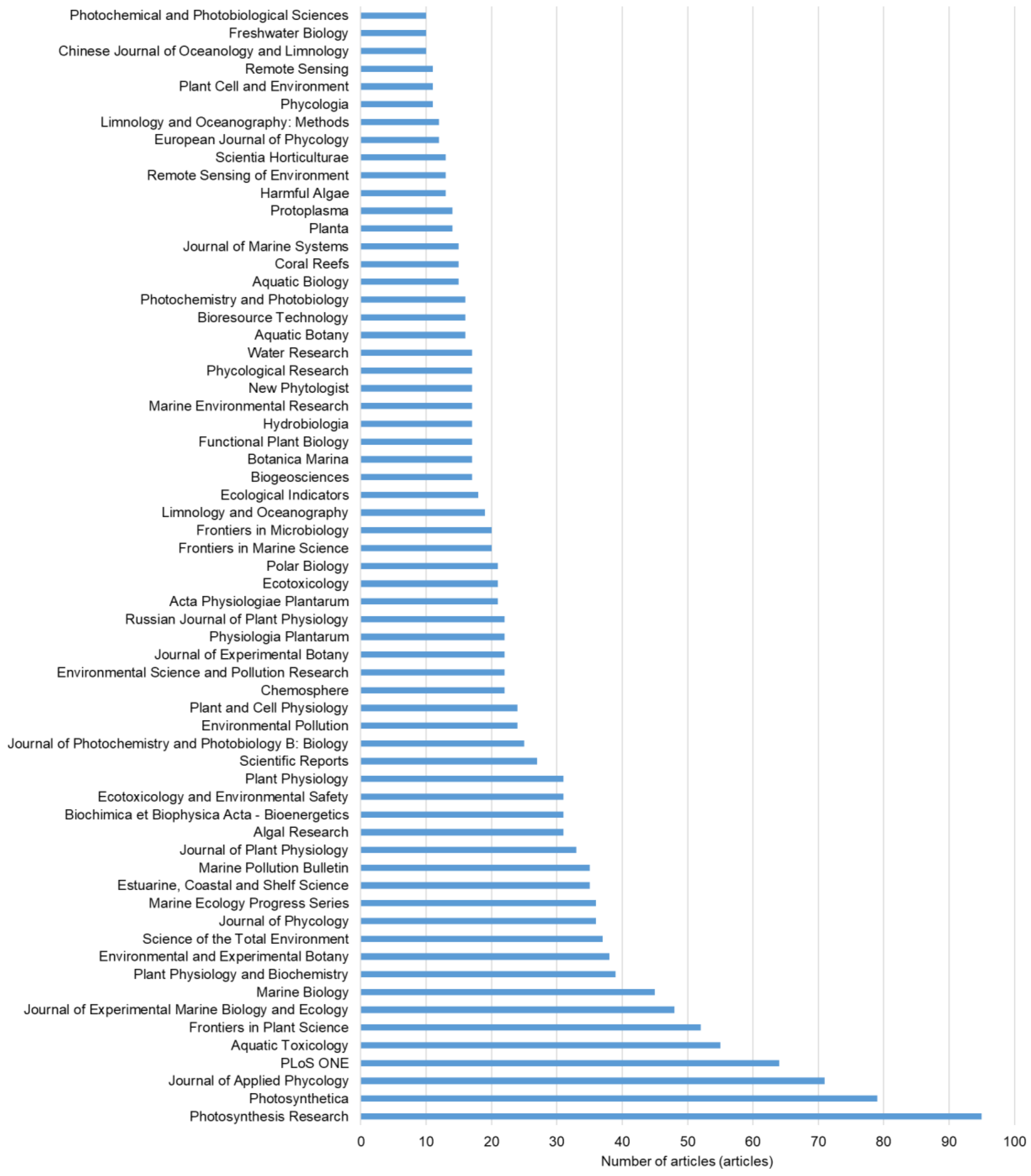


Figure S3: Scientific Journals with at least 10 published PAM articles.

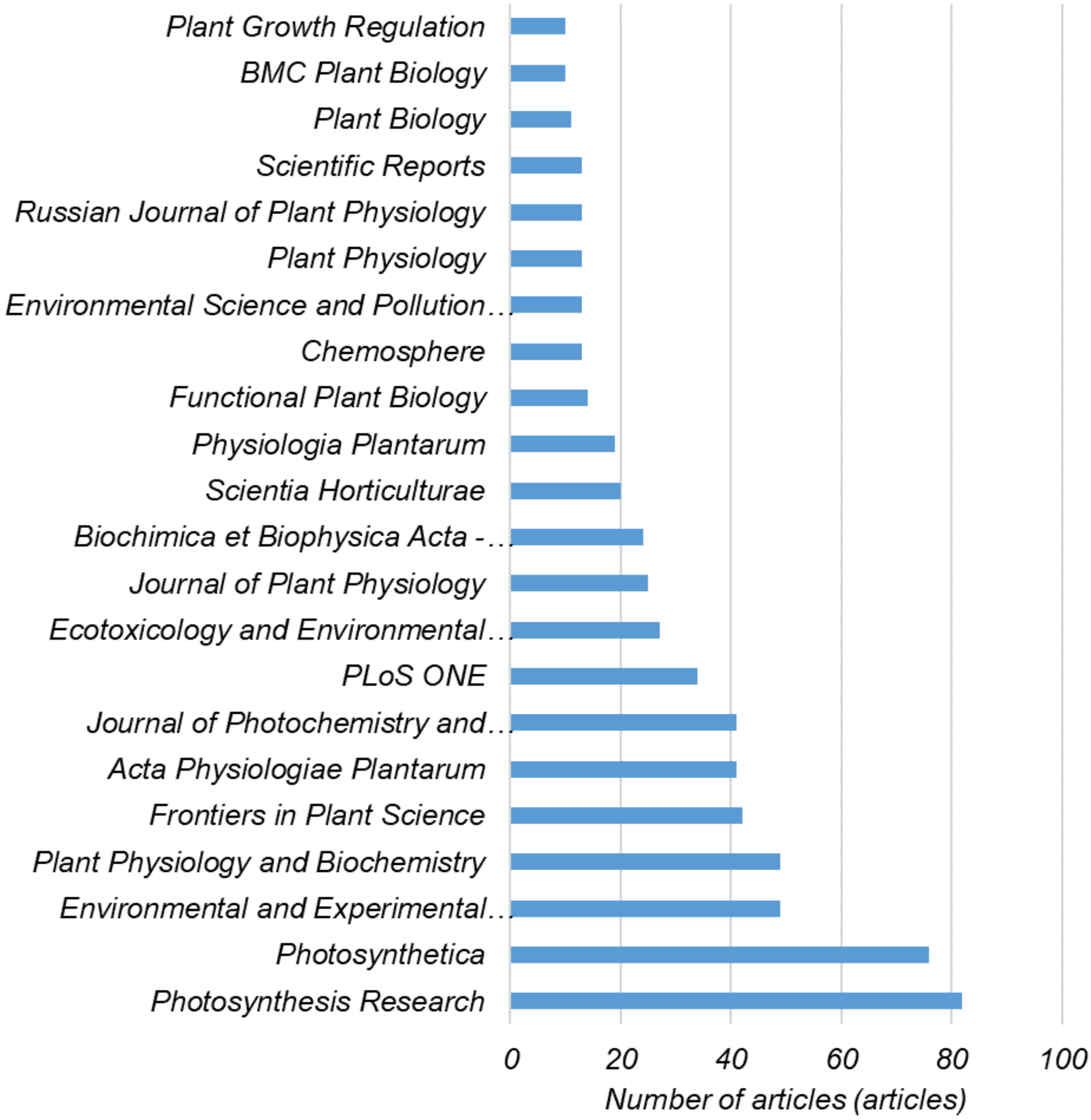


Figure S4: Scientific Journals with at least 10 published OJIP articles.

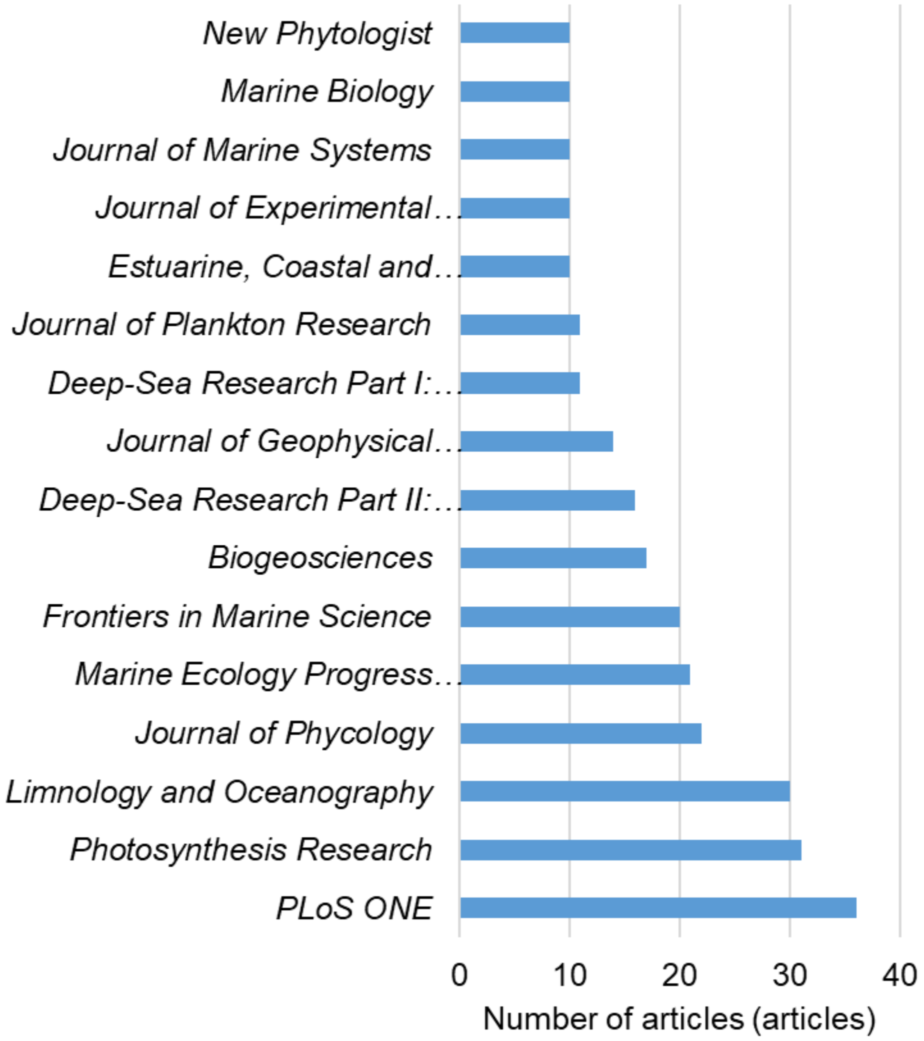


Figure S5: Scientific Journals with at least 10 published FRR articles.
